# LA-ICP-MS U-Pb zircon geochronology data of the Early to Mid-Miocene syn-extensional massive silicic volcanism in the Pannonian Basin (East-Central Europe)

**DOI:** 10.1016/j.dib.2018.05.013

**Published:** 2018-05-16

**Authors:** Réka Lukács, Marcel Guillong, Jakub Sliwinski, István Dunkl, Olivier Bachmann, Szabolcs Harangi

**Affiliations:** a MTA-ELTE Volcanology Research Group, 1117, Pázmány Péter sétány 1/C, Budapest, Hungary; b Institute of Geochemistry and Petrology, Department of Earth Sciences, ETH Zürich, Clausiusstrasse 25, 8092 Zürich, Switzerland; c Sedimentology & Environmental Geology, Geoscience Center, University of Göttingen, Goldschmidtstrasse 3, D-37077 Göttingen, Germany; d Department of Petrology and Geochemistry, Eötvös Loránd University, 1117, Budapest Pázmány Péter sétány 1/C, Budapest, Hungary

## Abstract

This article provides LA-ICP-MS in-situ U-Pb zircon dates performed on single crystals from dacitic to rhyolitic ignimbrites of the Bükkalja Volcanic Field (Hungary, East-Central Europe) temporally covering the main period of the Neogene silicic volcanic activity in the Pannonian Basin. The data include drift-corrected, alpha dose-corrected, Th-disequilibrium-corrected, and filtered data for geochronological use. The data presented in this article are interpreted and discussed in the research article entitled “Early to Mid-Miocene syn-extensional massive silicic volcanism in the Pannonian Basin (East-Central Europe): eruption chronology, correlation potential and geodynamic implications” by Lukács et al. (2018) [1].

## Specifications Table

**Table:** 

Subject area	*Earth Sciences*
More specific subject area	*Geochronology, Geochemistry*
Type of data	*Tables*
How data was acquired	*Laser-ablation inductively coupled mass spectrometry (LA-ICP-MS); Thermo Element XR Sector Field (SF)-ICP-MS with Resonetics Resolution 155 laser ablation system (ETH Zürich) and Thermo Element 2 SF-ICP-MS with Resonetics Resolution 155 laser ablation system (Göttingen University)*
Data format	*drift-corrected, filtered, alpha-dose and Th-disequilibrium corrected data in .xlsx format*
Experimental factors	*Zircon grains were extracted from bulk volcanic rocks (pumices, fiamme and bulk pyroclastic rocks)*
Experimental features	*Separated zircon grains were mounted in epoxy resin, polished and mapped by cathodoluminescence technique. Two samples were pre-treated by chemical abrasion before mounting*[2]
Data source location	*Bükkalja Volcanic Field, northern Hungary as reported in*Table 1.
Data accessibility	*Supplementary materials*

## Value of the data

• These data provide high-spatial resolution U-Pb dates of zircon grains based on ^206^Pb/^238^U isotope ratios of the silicic volcanic rocks from Bükkalja Volcanic Field (Hungary), allowing better constraints on eruption chronology.
• These new data can be compared to other in-situ zircon U-Pb dates in central Europe in order to correlate Miocene silicic pyroclastic horizons and ash-bearing sedimentary deposits in regional scale.
• These data are also valuable for detrital zircon geochronology in the Pannonian Basin system and other peri-Alpine basins to reveal redeposition of the pyroclastic material and help provenance determination.

## 1 Data

In this article, we report in-situ U-Pb zircon geochronological data from dacitic to rhyolitic pyroclastic rocks of the Bükkalja Volcanic Field, northern Hungary [1]. More than 1400 individual zircon in-situ analyses of single zircon grains (from 24 different samples) are listed. Data were obtained during 19 sessions along with common zircon reference materials (e.g. GJ-1, [3] 91500 [4]). The dataset contains the LA-ICP-MS raw and processed data.

## 2 Experimental design, materials and methods

### 2.1 Sample collection

Localities with GPS coordinates and lithology of the samples are shown in Table 1.

**Table 1:** Details of sample localities.

**Sample name**	**Locality, layer**	**GPS coordinates**	**Lithological name of analysed sample**
Harsány ignimbrite unit	Harsány ignimbrite unit		
Td-A; Td-A_CA	Tibolddaróc, layer A	47°55׳31.59"N, 20°37׳49.77"E	large pumice of rhyolite block-bearing lapilli tuff
Td-A_DX-46	Tibolddaróc, layer A	47°55׳31.59"N, 20°37׳49.77"E	large pumice of rhyolite block-bearing lapilli tuff
Tibolddaróc unit	Tibolddaróc unit		
Td-E	Tibolddaróc, layer E	47°55׳36.64"N, 20°37׳55.19"E	rhyolite lapilli tuff
Demjén ignimbrite unit	Demjén ignimbrite unit		
Td-H; Td-H_CA	Tibolddaróc, layer H	47°55׳33.45"N, 20°37׳55.55"E	rhyolite lapilli-bearing tuff
Td-H_DX-47	Tibolddaróc, layer H	47°55׳33.45"N, 20°37׳55.55"E	rhyolite lapilli-bearing tuff
FN-1	Felnémet, old quarry	47°56׳0.09"N, 20°22׳58.88"E	rhyolite lapilli tuff
DEMNE-1	Demjén, Nagyeresztvény quarry	47°50׳1.51"N, 20°20׳37.19"E	rhyolite lapilli tuff
DEMNE-1_DX-48	Demjén, Nagyeresztvény quarry	47°50׳1.51"N, 20°20׳37.19"E	rhyolite lapilli tuff
DEMSPA	Demjén, Spa side	47°50׳16.54"N, 20°20׳20.78"E	rhyolite lapilli tuff
DEMSPA_DX-7	Demjén, Spa side	47°50׳16.54"N, 20°20׳20.78"E	rhyolite lapilli tuff
TAR-3	Tar, Fehérkő quarry	47°57׳9.88"N, 19°45׳46.45"E	pumice of lapillituff
Td-L	Tibolddaróc, layer L	47°55׳39.01"N, 20°37׳59.68"E	rhyolite accretionary lapilli-bearing tuff
Bogács unit	Bogács unit		
Td-S	Tibolddaróc, layer M (UMPU)	47°55׳41.49"N, 20°37׳58.37"E	ablack scoria clasts of dacite scoria-bearing lapillit tuff
Td-Hk1_CA	Tibolddaróc, layer M (UMPU)	47°55׳41.49"N, 20°37׳58.37"E	bgrey scoria clasts of dacite scoria-bearing lapillit tuff
Td-H2N; Td-H2N_CA	Tibolddaróc, layer M (UMPU)	47°55׳41.49"N, 20°37׳58.37"E	bgrey scoria clasts of dacite scoria-bearing lapillit tuff
Td-Fi; Td-Fi_CA	Tibolddaróc, old quarry, layer M (LWPU)	47°55׳48.14"N, 20°37׳56.92"E	cfiamme clasts of dacite fiamme-bearing lapillit tuff
CSF-KEV	Cserépfalu, Geosite	47°56׳34.42"N, 20°32׳25.98"E	dacite scoria-bearing lapilli tuff
CSF-KEV_DX-05	Cserépfalu, Geosite	47°56׳34.42"N, 20°32׳25.98"E	dacite scoria-bearing lapilli tuff
Mangó ignimbrite unit	Mangó ignimbrite unit		
EG-2	Eger, Tihamér-quarry (upper, active)	47°53׳8.04"N, 20°24׳14.38"E	rhyolite lapilli tuff
EG-2_DX-56	Eger, Tihamér-quarry (upper, active)	47°53׳8.04"N, 20°24׳14.38"E	rhyolite lapilli tuff
SZOM	Szomolya, fairy chimneys	47°53׳29.74"N, 20°28׳40.71"E	rhyolite lapilli tuff
SZOM_DX-49	Szomolya, fairy chimneys	47°53׳29.74"N, 20°28׳40.71"E	rhyolite lapilli tuff
Mt-1	Cserépváralja, Mangó-tető	47°55׳36.15"N, 20°34׳17.11"E	large pumice of rhyolite block-bearing lapilli tuff
DEMHAN1	Demjén, Hangács, old quarry	47°50׳32.89"N, 20°20׳21.98"E	rhyolite lapilli tuff
CSkly1	Cserépfalu, Kőporlyuk	47°56׳40.26"N, 20°32׳30.01"E	rhyolite accretionary lapilli bearing tuff
CsO1	Cserépfalu, Ördögcsúszda	47°57׳34.69"N, 20°32׳47.72"E	large pumice of rhyolite block-bearing lapilli tuff
CsTb1	Cserépfalu, Túr-bucka	47°57׳40.11"N, 20°32׳33.81"E	rhyolite lapilli tuff
S_DX-03	Sály, Latorút	47°58׳1.07"N, 20°38׳49.81"E	rhyolite lapilli tuff
K_DX-04	Kács, templom tér	47°57׳25.39"N, 20°36׳54.66"E	rhyolite lapilli tuff
Eger ignimbrite unit	Eger ignimbrite unit		
EG-1	Eger, Tihamér old quarry (lower)	47°53׳7.19"N, 20°24׳0.63"E	rhyolite lapilli tuff
EG-1_DX-55	Eger, Tihamér old quarry (lower)	47°53׳7.19"N, 20°24׳0.63"E	rhyolite lapilli tuff
Csv-2	core sample from 240 to 243 m of Csv-2 drilling	47°55׳ 18,43"N, 20°33׳ 59,77"E	rhyolite lapilli tuff

raw data of Td-E, FN-1 and DEMNE-1 were published in [5].

a Black coloured scoria clast of UMPU [6].

b Grey coloured scoria clast of UMPU [6].

c A-fiamme type clast of LWPU [6].

### 2.2 Sample preparation

Zircon crystals were separated from the 63 to 125 µm size fraction of rock samples by standard gravity and magnetic separation methods. The amount of xenocrystic zircons was minimized by separating zircon grains solely from pumice clasts of the pyroclastic rock (when available), while in case of lapilli tuff samples we attempted to remove all lithic fragments before zircon separation.

In order to minimize the effects of lead loss, chemical abrasion (CA; [2]) was employed on two aliquots of zircons analysed by LA ICP-MS (TD-A_CA; TD-H_CA). Zircon grains of each sample were loaded into quartz crucibles and annealed in a high temperature furnace (900 °C) for 48 h. The zircons were transferred from the quartz crucibles into 3 ml Savillex PFA Hex beakers and concentrated HF + trace HNO_3_ was added. The beakers were placed in a high pressure Parr bomb and the zircons were etched at 180 °C for 12–15 h. The zircons were rinsed with H_2_O and acetone before being fluxed for 12 h in 6 N HCl at ~ 85 °C. The zircons were rinsed in H_2_O and washed with acetone.

The separated zircon grains were mounted in 1 in. epoxy resin mount and polished to a 1 µm finish. Before dating, zircons were checked by optical microscopic and cathodoluminescence (CL) imaging. CL imaging was produced using an AMRAY 1830 SEM equipped with GATAN MiniCL and 3 nA, 10 kV setup at the Department of Petrology and Geochemistry, Eötvös University, Hungary and a JEOL JXA 8900 electron microprobe with 10 kV setup at the University of Göttingen.

### 2.3 LA-ICP-MS analyses

Analyses were performed in two laboratories: Department of Earth Sciences, ETH Zürich and GÖochron Laboratories, University of Göttingen. Analytical setups of the laboratories are presented in Table 2, Table 3.

**Table 2:** LA-ICP-MS U-Pb analysis performed at ETH Zürich.

**Laboratory name**	**Department of Earth Sciences, ETH Zürich**
**Laser ablation system**	
Make, Model & type	ASI Resolution 155
Ablation cell & volume	Laurin Technics 155, constant geometry, aerosol dispersion volume < 1 cm^3^
Laser wavelength	193 nm
Pulse width	25 ns
Fluence	~ 2 J cm^-2^
Repetition rate	5 Hz
Spot size	30 µm
Ablation rate	~ 75 nm pulse^-1^
Sampling mode/pattern	Single hole drilling, 5 cleaning pulses
Carrier gas	100% He
Ablation duration	40 s
Cell carrier gas flow	0.7 l/min
**ICP-MS Instrument**	
Make, Model & type	Thermo Element XR SF-ICP-MS
Sample introduction	Ablation aerosol only, squid aerosol homogenization device
RF power	1500 W
Make-up gas flow	~ 0.95 l/min Ar (gas mixed to He carrier inside ablation cell funnel)
Detection system	Single detector triple mode SEM, analogue, Faraday
Masses measured	202, 204, 206, 207, 208, 232, 235, 238 amu
Integration time per peak	12 ms (masses 202, 204), 20 ms (masses 208, 232, 235, 238), 40 ms (masses 206, 207)
Total integration time per reading	0.202 s
Dead time	8 ns
Typical oxide rate (ThO/Th)	0.18%
Typical doubly charged rate (Ba^++^/Ba^+^)	3.5%
**Data Processing**	
Gas blank	10 s prior to each ablation spot
Calibration strategy	GJ-1 used as primary calibration material in all sessions except for the two sessions with chemically abraded samples where chemically abraded GJ-1 (GJ-1_CA) was used as calibration reference material along with chemically abraded validation reference materials (Temora2, 91500, OD-3)
	Validation reference materials used in sessions:
	session 140614: Plešovice, 91500, Temora2, LG_0302
	session 140815: Plešovice, 91500, Temora2, OD-3
	session 140204b, 140205: Plešovice, 91500, Temora2
	session 150323, 150324, 150327: Plešovice, 91500, Temora2, OD-3
	session 160409p2: Plešovice, 91500, AUSZ7-1, AUSZ7-5
	References:
	Plešovice [7], [8], 91500 [4], [8], Temora2 [9], OD-3 [10], AUSZ7-1 [11] and AUSZ7-5 [12], LG_0302 (pers. comm. von Quadt, 2017)
Reference Material info	GJ-1 ^206^Pb/^238^U 0.09761 ± 0.0002 (weighted mean of ID-TIMS analysis ± 2*σ*, [3])
Data processing package used	IOLITE v2.5, v3.4 [13], [14] with VizualAge [15]
Mass discrimination	Mass bias correction for all ratios normalized to calibration reference material
Common Pb correction	No common-Pb correction applied
Uncertainty level & propagation	Ages are quoted at 2 SE absolute, propagation is by quadratic addition. Reproducibility of reference material uncertainty (i.e. external uncertainty) is propagated.

**Table 3:** LA-ICP-MS U-Pb analysis performed at University of Göttingen.

**Laboratory name**	**GÖochron Laboratories, University of Göttingen**
**Laser ablation system**	
Make, Model & type	ASI Resolution 155
Ablation cell & volume	Laurin Technics 155, constant geometry, aerosol dispersion volume < 1 cm^3^
Laser wavelength	193 nm
Pulse width	25 ns
Fluence	~ 2 J cm^-2^
Repetition rate	5 Hz
Spot size	33 µm
Sampling mode	Single hole drilling, 2 cleaning pulses
Carrier gas	100% He
Ablation duration	20 s
Cell carrier gas flow	0.7 l/min
**ICP-MS Instrument**	
Make, Model & type	Thermo Element 2 SF-ICP-MS
Sample introduction	Ablation aerosol only, squid aerosol homogenization device
RF power	1400 W
Make-up gas flow	~ 1 l/min Ar (gas mixed to He carrier inside ablation cell funnel)
Detection system	Single detector dual mode SEM, analog
Masses measured	202, 204, 206, 207, 208, 232, 235, 238 amu
Integration time per peak	10 ms (masses 232, 238), 15 ms (masses 202, 204, 235), 30 ms (mass 208), 60 ms (mass 206), 100 ms (mass 207)
Total integration time per reading	255 ms
Dead time	21 ns
Typical oxide rate (UO/U)	0.04%
Typical doubly charged rate (Ba^++^/Ba^+^)	N/A
**Data Processing**	
Gas blank	9 s prior to each ablation spot
Calibration strategy	GJ-1 used as calibration reference material in all sessions (9)
	Validation reference materials used in these sessions:
	91500 [4], FC-1 [16]
Reference Material info	GJ-1 ^206^Pb/^238^U: 0.09761 ± 0.00006 (weighted mean of ID-TIMS analysis ± 2*σ*, [3])
Data processing package used	UranOS 2.08a [17]http://www.sediment.uni-goettingen.de/staff/dunkl/software/uranos.html
Mass discrimination	Mass bias correction for all ratios normalized to calibration reference material
Common Pb correction	No common Pb correction applied
Uncertainty level & propagation	Ages are quoted at 2 SE absolute, propagation is by quadratic addition. Reproducibility of reference material uncertainty is propagated.

### 2.4 Data handling

We filtered out the data that was > 10% discordant determined by the following equation:

Discordance=100*1−P206bU238AgeP207bU235Age

Validation reference materials were used to correct for alpha dose-dependent age offsets in non-CA treated zircons [18], [19]. In short, accumulation of radiation damage in a zircon weakens the matrix, increasing the ablation rate and the effects of laser-induced elemental fractionation. This in turn imparts a differential downhole fractionation curve between calibration and validation reference materials, making low-dose (i.e. young and low-U) zircons appear anomalously young following downhole fractionation correction. This effect can be mitigated by modelling the dependence of age offset on total radiation dose, calculated from sample age and concentrations of U and Th [20]. Because thermal annealing repairs some matrix radiation damage [18], [19], it is important that samples and reference materials are either all thermally annealed, or all not thermally annealed. The age offset vs. alpha dose model also become inaccurate if some zircons have experienced natural thermal annealing through contact metamorphism or burial. However, given that the samples in question are young and show no signs of contact metamorphism, we can exclude this possibility. Possible natural annealing of zircons was also excluded based on Raman spectroscopy (i.e. alpha dose concentrations and Raman band parameters of zircon crystals are in agreement; [21]). At ETH Zürich, the relationship between age offsets and alpha dose concentrations were modelled in each session and this model was used to calculate the alpha-dose corrected ages. At Göttingen University, measurements were alpha dose corrected based on a global model of validation reference material measurements of all sessions between 2014 and 2017. In both cases, Th disequilibrium correction was performed after alpha dose-correction using the algorithm of [22], assuming a constant Th/U partition coefficient ratio of 0.33 ± 0.063 (1*σ*) [23].
